# Supplementary material for: Diaphanous-related formin mDia2 regulates beta2 integrins to control hematopoietic stem and progenitor cell engraftment
Source: Nat Commun. 2020 Jun 23;11:3172. doi: 10.1038/s41467-020-16911-4 (PMC7311390; doi:10.1038/s41467-020-16911-4)
Supplement: Supplementary file 1 — Supplementary Information [file 41467_2020_16911_MOESM1_ESM.pdf]

Supplementary Information

Diaphanous-related formin mDia2 regulates beta2 integrins to control hematopoietic stem and progenitor cell engraftment

Yang Mei[1,2 *], Xu Han[1,2], Yijie Liu[1,2], Jing Yang[1,2], Ronen Sumagin[1,2], and Peng Ji[1,2 *]

1. Department of Pathology, Feinberg School of Medicine, Northwestern University, Chicago, IL, 60611 USA

2. The Robert H. Lurie Comprehensive Cancer Center, Northwestern University, Chicago, IL, 60611 USA

* Correspondence:yang.mei@northwestern.edu or peng-ji@fsm.northwestern.edu

Key words: Hematopoietic stem and progenitor cells, engraftment, Diaphanous-related formins, mDia2, trans-endothelial migration, serum response factor, beta2 integrins, CD11b, CD18

Supplementary Figures

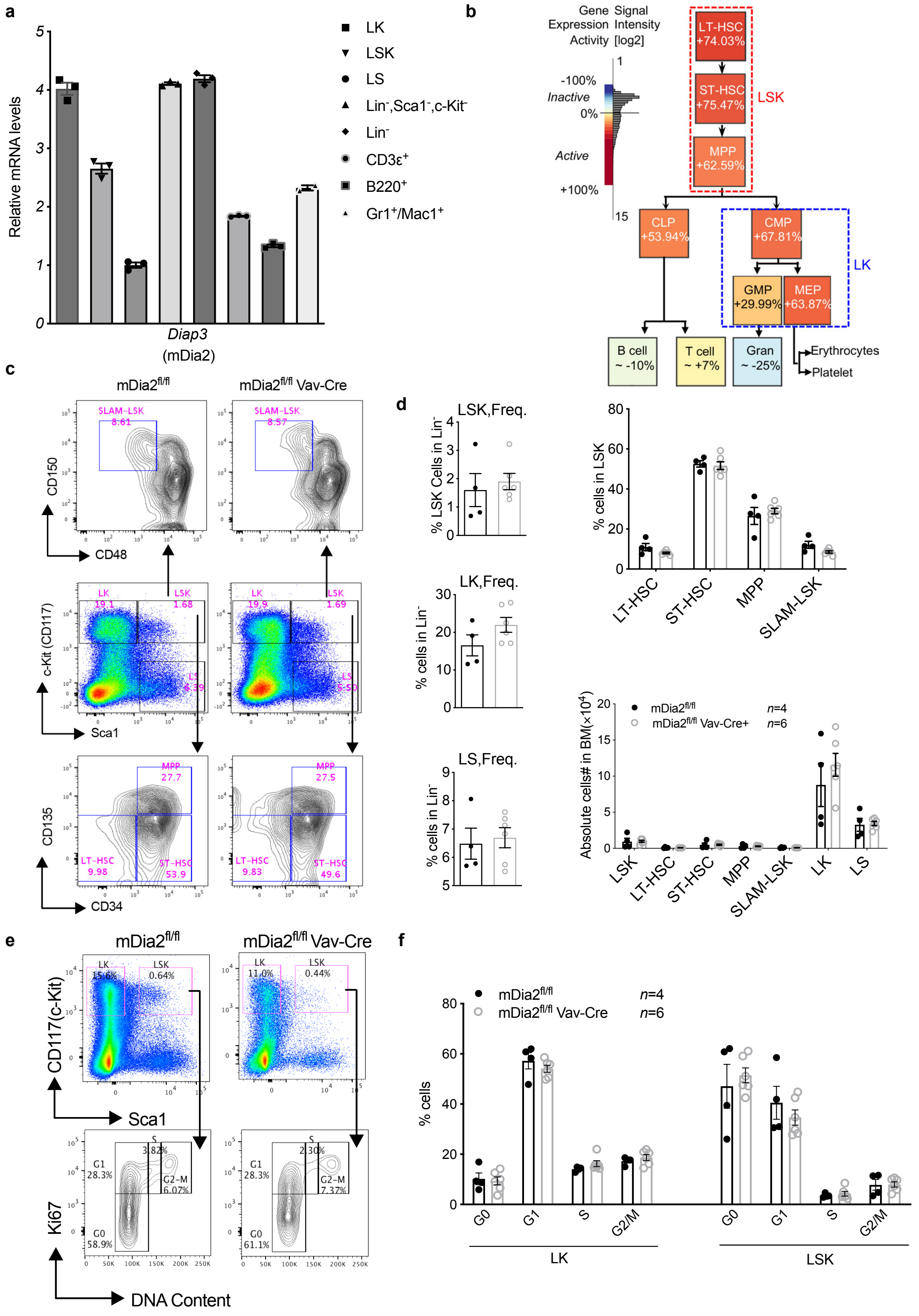

Supplementary Figure 1. Loss of mDia2 doesn't affect the compositions of HSPCs at steady state (**a**) Quantitative real time PCR analyses of the mRNA levels of mouse *Diap3* in the indicated lineages. 18S ribosomal RNA was used as an internal control. The experiment was repeated in triplicate. (**b**) Highly enriched *Diap3* expression in HSPCs obtained from Gene Expression Commons database [1]. (**c**) Representative flow cytometry plots for the gating of different HSPC populations in the bone marrow lineage negative cells from mDia2$^{fl/fl}$ Vav-Cre and mDia2$^{fl/fl}$ control mice at 3-month old. (**d**) Quantitative analyses of the percentage and absolute cell number of indicated HSPC populations in c. (**e**) Representative flow cytometry plots of cell cycle profiles of LSK cells from the indicated mice as in c. (**f**) Quantitative analyses cell cycle profiles of the LK and LSK cells from the bone marrow of indicated mice at 3-month old. n=4 mice in mDia2$^{fl/fl}$ group, n=6 mice in mDia2$^{fl/fl}$ Vav-Cre group in d and f. Data is presented as mean ± SEM. *$p < 0.05$, **$p < 0.01$, ***$p < 0.001$, **** $p<0.0001$. Two-tailed unpaired Student's t test was used to generate the p values.

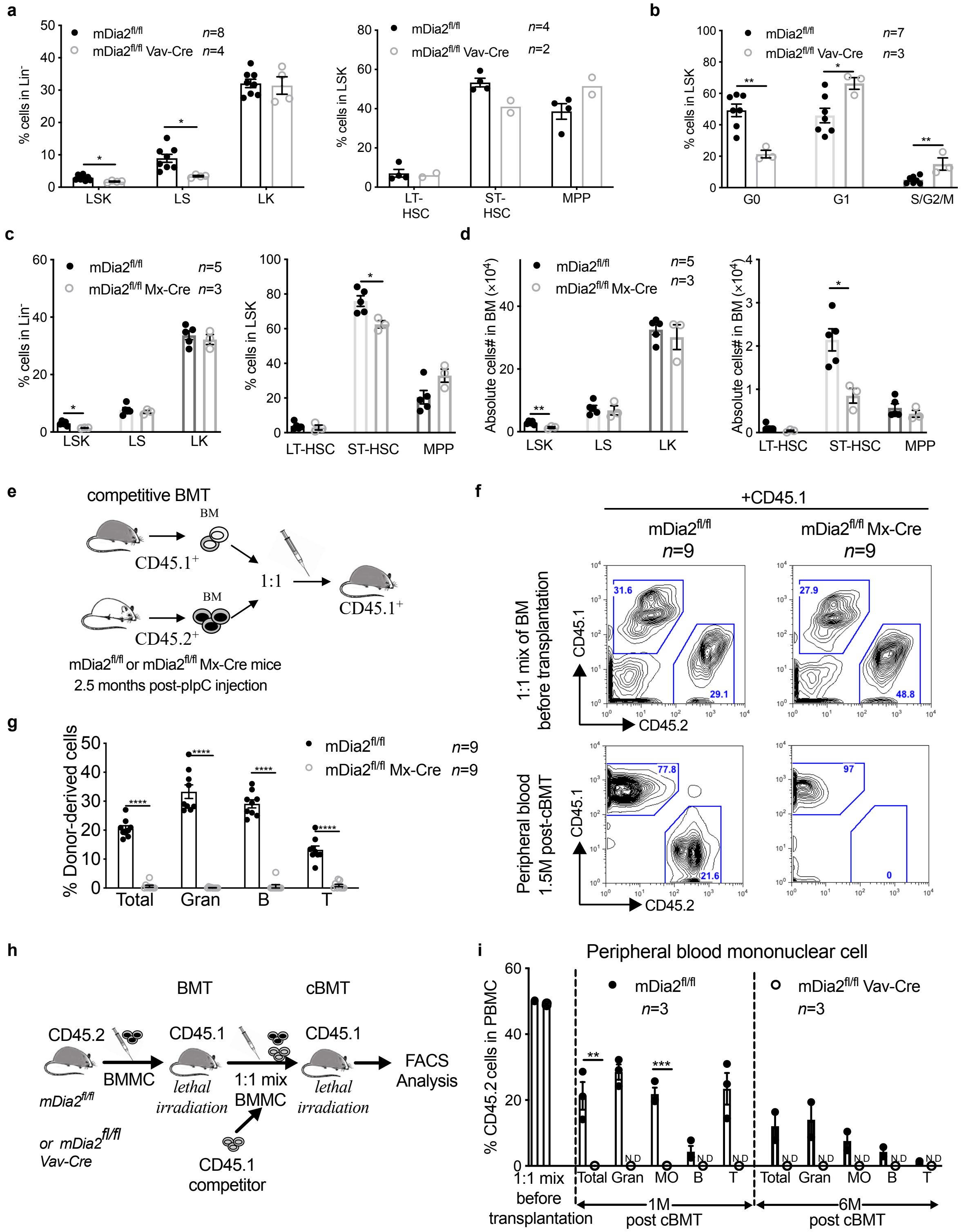

a
% cells in Lin⁻
mDia2fl/fl n=8
mDia2fl/fl Vav-Cre n=4
LSK
LS
LK
% cells in LSK
mDia2fl/fl n=4
mDia2fl/fl Vav-Cre n=2
LT-HSC
ST-HSC
MPP
b
% cells in LSK
mDia2fl/fl n=7
mDia2fl/fl Vav-Cre n=3
G0
G1
S/G2/M
c
% cells in Lin⁻
mDia2fl/fl n=5
mDia2fl/fl Mx-Cre n=3
LSK
LS
LK
% cells in LSK
LT-HSC
ST-HSC
MPP
d
Absolute cells# in BM (×10⁴)
mDia2fl/fl n=5
mDia2fl/fl Mx-Cre n=3
LSK
LS
LK
Absolute cells# in BM (×10⁴)
LT-HSC
ST-HSC
MPP
e
competitive BMT
BM
CD45.1+
1:1
CD45.1+
BM
CD45.2+
mDia2fl/fl or mDia2fl/fl Mx-Cre mice
2.5 months post-pIpC injection
f
+CD45.1
mDia2fl/fl n=9
mDia2fl/fl Mx-Cre n=9
1:1 mix of BM before transplantation
CD45.1
CD45.2
31.6
29.1
27.9
48.8
Peripheral blood 1.5M post-cBMT
77.8
21.6
97
0
g
% Donor-derived cells
mDia2fl/fl n=9
mDia2fl/fl Mx-Cre n=9
Total
Gran
B
T
h
BMT
cBMT
CD45.2
CD45.1
CD45.1
BMMC
lethal irradiation
1:1 mix BMMC
lethal irradiation
FACS Analysis
mDia2fl/fl
or mDia2fl/fl Vav-Cre
CD45.1 competitor
i
Peripheral blood mononuclear cell
% CD45.2 cells in PBMC
mDia2fl/fl n=3
mDia2fl/fl Vav-Cre n=3
1:1 mix before transplantation
Total Gran MO B T
1M post cBMT
Total Gran MO B T
6M post cBMT
N.D

Supplementary Figure 2. Defects of competitive engraftment ability in mDia2 deficient HSPCs (**a**) The percentages of bone marrow HSPCs in wild type recipient mice 10 months after transplantation with 2 x 10^6 control or mDia2$^{fl/fl}$Vav-Cre BMMCs. Left panel, *n*=8 mice in mDia2$^{fl/fl}$ group, *n*=4 mice in mDia2$^{fl/fl}$ Vav-Cre group. Rght panel, *n*=4 mice in mDia2$^{fl/fl}$ group, *n*=2 mice in mDia2$^{fl/fl}$ Vav-Cre group. (**b**) Cell cycle profile of LSK cells from a. *n*=7 mice in mDia2$^{fl/fl}$ group, *n*=3 mice in mDia2$^{fl/fl}$ Vav-Cre group. (**c**) The percentages of bone marrow HSPCs in wild type recipient mice 10 months after transplantation with 2 x 10^6 control or mDia2$^{fl/fl}$Mx1-Cre BMMCs. (**d**) The absolute number of the indicated HSPCs in c. *n*=5 mice in mDia2$^{fl/fl}$ group, *n*=3 mice in mDia2$^{fl/fl}$ Vav-Cre group for c and d. (**e**) Experimental design of competitive bone marrow transplantation with pIpC treated mDia2$^{fl/fl}$ and mDia2$^{fl/fl}$ Mx-Cre mice. (**f**) Chimerism studies in the peripheral blood 1.5 months after transplantation as in e. (**g**) Quantitative analyses of the indicated lineages in e. *n*=9 mice in each group. (**h**) Experimental design of secondary competitive transplantation using 2×10^6 BMMCs from the indicated recipients after primary transplantation with an equal number of wild-type CD45.1 BMMCs. cBMT represents competitive bone marrow transplantation. (**i**) Peripheral blood chimerism analyses were performed 1 month after competitive transplantation in h. *n*=3 mice in each group. Error bars represent the SEM of the mean. *p < 0.05, **p < 0.01, ***p < 0.001, ****p < 0.0001. Two-tailed unpaired student's t test was used to generate the *p* values.

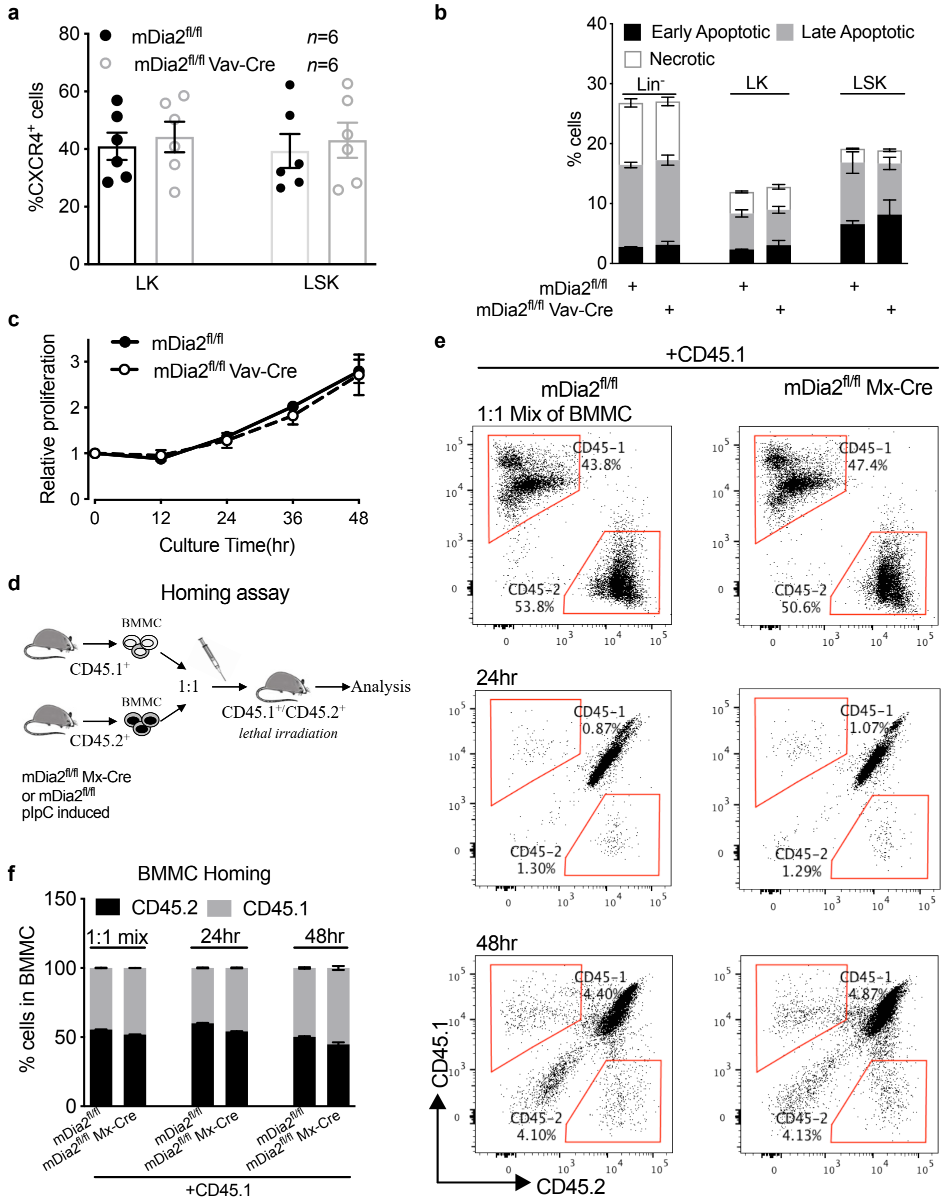
a
mDia2fl/fl n=6
mDia2fl/fl Vav-Cre n=6
%CXCR4+ cells
LK
LSK
b
Early Apoptotic
Late Apoptotic
Necrotic
Lin-
LK
LSK
% cells
mDia2fl/fl
mDia2fl/fl Vav-Cre
c
mDia2fl/fl
mDia2fl/fl Vav-Cre
Relative proliferation
Culture Time(hr)
d
Homing assay
BMMC
CD45.1+
CD45.2+
1:1
Analysis
CD45.1+/CD45.2+
lethal irradiation
mDia2fl/fl Mx-Cre
or mDia2fl/fl
pIpC induced
e
+CD45.1
mDia2fl/fl
mDia2fl/fl Mx-Cre
1:1 Mix of BMMC
CD45-1 43.8%
CD45-2 53.8%
CD45-1 47.4%
CD45-2 50.6%
24hr
CD45-1 0.87%
CD45-2 1.30%
CD45-1 1.07%
CD45-2 1.29%
48hr
CD45-1 4.40%
CD45-2 4.10%
CD45-1 4.87%
CD45-2 4.13%
CD45.1
CD45.2
f
BMMC Homing
CD45.2
CD45.1
1:1 mix
24hr
48hr
% cells in BMMC
mDia2fl/fl
mDia2fl/fl Mx-Cre
+CD45.1

Supplementary Figure 3. Loss of mDia2 doesn't affect HSPC localization to the BM vasculature (**a**) Quantitative analyses of the expression levels of CXCR4 by flow cytometry in the bone marrow LK and LSK cells from the indicated mice. *n*=6 in each group. (**b**) Quantitative analyses of the percentage of apoptotic cells in bone marrow LK and LSK cells from the indicated mice. *n*=3 in each group. (**c**) Relative proliferation rate of the bone marrow c-kit+ HSPCs from the indicated mice during ex vivo expansion. *n*=6 in mDia2$^{fl/fl}$ group, *n*=4 in mDia2$^{fl/fl}$ Vav-Cre group. (**d**) Experimental design of the homing assay. 2x10^{6} BMMCs from pIpC treated mDia2$^{fl/fl}$ or mDia2$^{fl/fl}$ Mx-Cre mice mixed with an equal number of CD45.1+ BMMCs from the wild type counterparts were injected into lethally irradiated wild type recipient mice. After 24 or 48 hours, BMMCs were obtained from the recipients for chimerism studies by flow cytometry. (**e**) Representative flow cytometry plots as described in d at 24 and 48 hours. (**f**) Quantitative analyses of e. *n*=3 in each group. All the error bars represent the SEM of the mean.

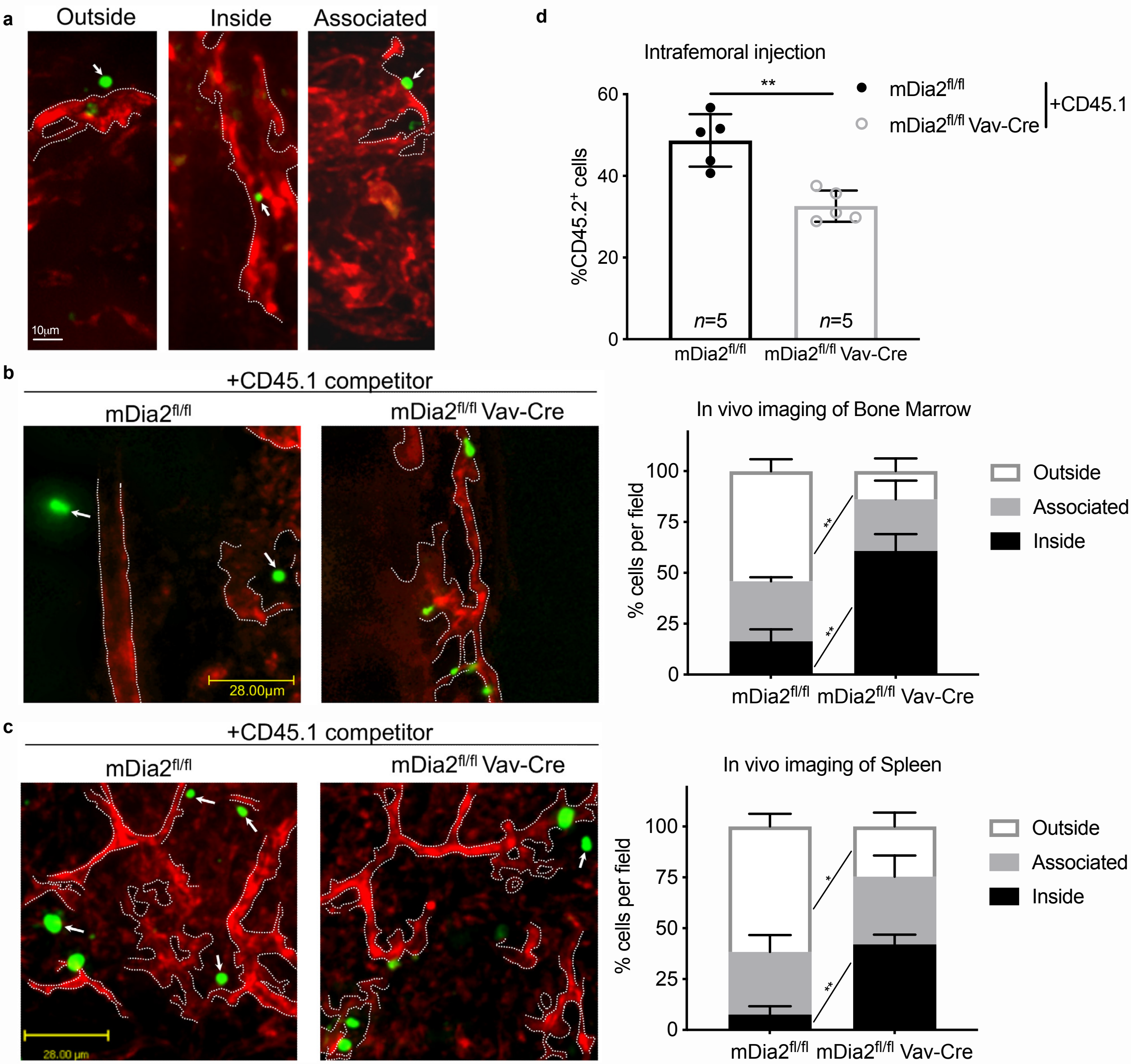
a
Outside
Inside
Associated
10μm
b
+CD45.1 competitor
mDia2fl/fl
mDia2fl/fl Vav-Cre
28.00μm
c
+CD45.1 competitor
mDia2fl/fl
mDia2fl/fl Vav-Cre
28.00 μm
d
Intrafemoral injection
%CD45.2+ cells
60
40
20
0
**
mDia2fl/fl
mDia2fl/fl Vav-Cre
+CD45.1
n=5
n=5
mDia2fl/fl
mDia2fl/fl Vav-Cre
In vivo imaging of Bone Marrow
% cells per field
100
75
50
25
0
**
**
Outside
Associated
Inside
mDia2fl/fl
mDia2fl/fl Vav-Cre
In vivo imaging of Spleen
% cells per field
100
75
50
25
0
*
**
Outside
Associated
Inside
mDia2fl/fl
mDia2fl/fl Vav-Cre

Supplementary Figure 4. Loss of mDia2 compromises HSPC's trans-endothelial migration (**a**) Representative images of HSPC association with the vessels. Arrows point to the transplanted HSPCs. Dashed white lines outline the vessels. (**b**) *In vivo* imaging of fluorescently labeled lineage negative donor cells in the femur of the wild type mice 4 hours after non-irradiated competitive transplantation with equal number ($9x10^5$) of non-fluorescently labeled CD45.1+ cells. Red, CD31+ endothelial cells. Green, CD45.2+ donor cells. Dashed white lines outline the vessels. The relative position of the indicated donor cells to the outlined CD31+ lining sinusoids or arteries were quantified (right). N=39 cells from 6 random fields in mDia2$^{fl/fl}$ group. N=30 cells from 5 random fields in mDia2$^{fl/fl}$Vav-Cre group. (**c**) Same as b except the spleen was analyzed. N=30 cells from 3 random fields in mDia2$^{fl/fl}$ group. N=24 cells from 3 random fields in mDia2$^{fl/fl}$Vav-Cre group. (**d**) $2x10^6$ mDia2$^{fl/fl}$ or mDia2$^{fl/fl}$ Vav-Cre CD45.2+ BMMCs were mixed with equal number of wild-type CD45.1+ competitive BMMCs and transplanted into lethally irradiated wild type mice (CD45.1+, n=5 mice in each group) through intrafemoral route. Bone marrow chimerism studies were performed 4 weeks after transplantation using flow cytometric analyses. Error bars represent the SEM of the mean. *p < 0.05, **p < 0.01. Two-tailed unpaired student's t test was used to generate the p values.

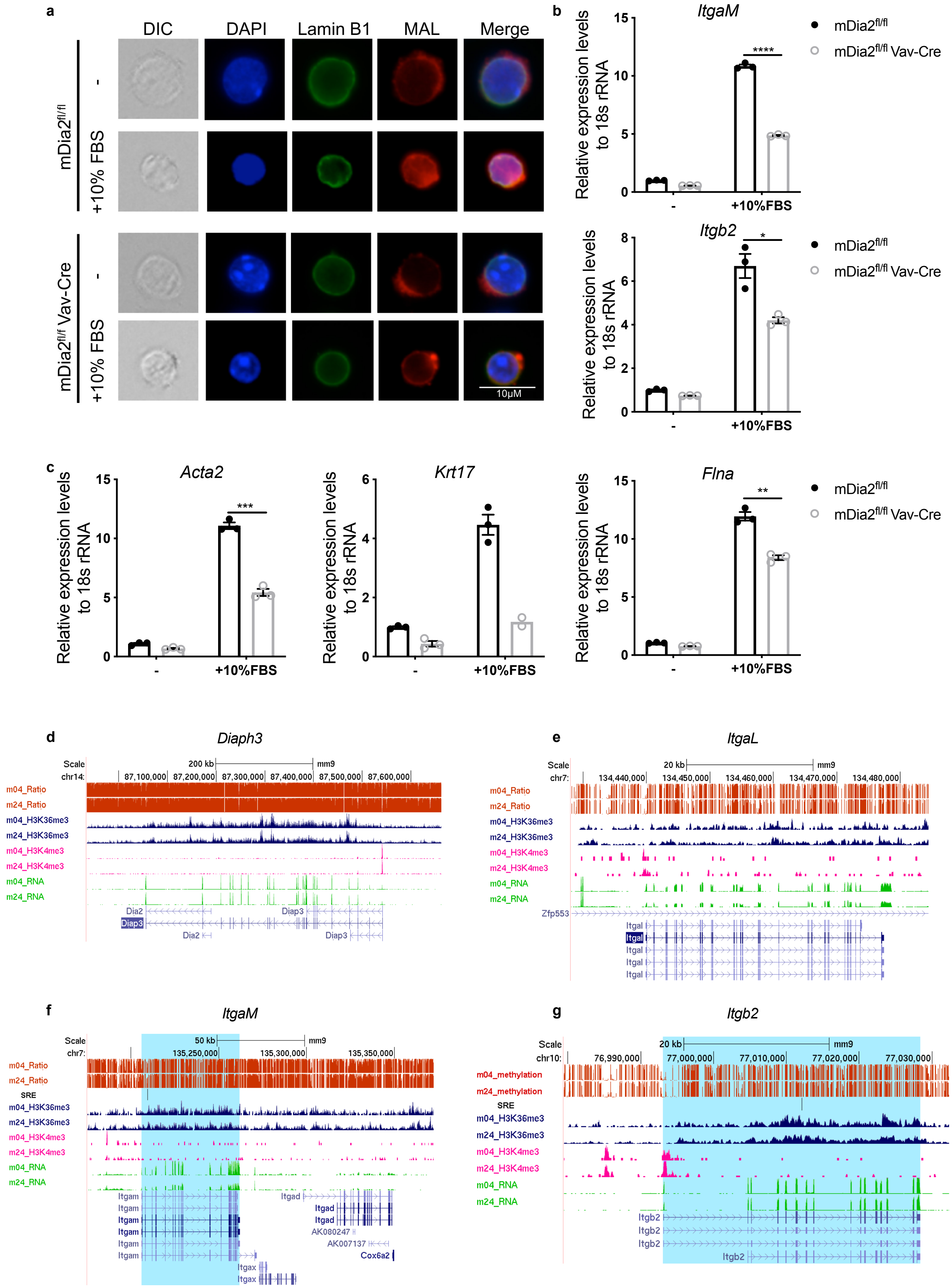
a
DIC
DAPI
Lamin B1
MAL
Merge
mDia2fl/fl
-
+10% FBS
mDia2fl/f Vav-Cre
10μM
b
ItgaM
Itgb2
Relative expression levels to 18s rRNA
+10%FBS
mDia2fl/fl
mDia2fl/fl Vav-Cre
c
Acta2
Krt17
Flna
d
Diaph3
e
ItgaL
f
ItgaM
g
Itgb2
m04_Ratio
m24_Ratio
m04_H3K36me3
m24_H3K36me3
m04_H3K4me3
m24_H3K4me3
m04_RNA
m24_RNA
m04_methylation
m24_methylation
SRE

Supplementary Figure 5. mDia2-SRF signaling is involved in the regulation of HSPC engraftment (**a**) Immunofluorescence analyses of MAL and lamin B1 in c-Kit+ HSPCs from the indicated mice incubated with or without FBS for 15 minutes. Repeated three times with similar results. (**b**) Relative mRNA expression levels of *ItgaM* and *Itgb2* in c-Kit+ HSPCs from the indicated mice incubated with or without FBS for 15 minutes. (**c**) Same as b except the indicated genes were analyzed. The experiments in b and c were performed in triplicate. (**d-g**) UCSC browser track showing the expression (green, RNA-Seq), DNA methylation (red), H3K36me3 peaks (dark blue), and H3K4me3 for transcription start sites (TSS, pink) of actively transcribed regions of the indicated genes in young (4 months) and old (24 months) HSCs. Data were obtained from aging HSC Epigenome [2]. Blue shaded regions represent the genomic regions of *ItgaM* and *Itgb2*. Error bars represent the SEM of the mean. *$p < 0.05$, **$p < 0.01$, ***$p < 0.001$, ****$p < 0.0001$. Two-tailed unpaired student's t test was used to generate the p values.

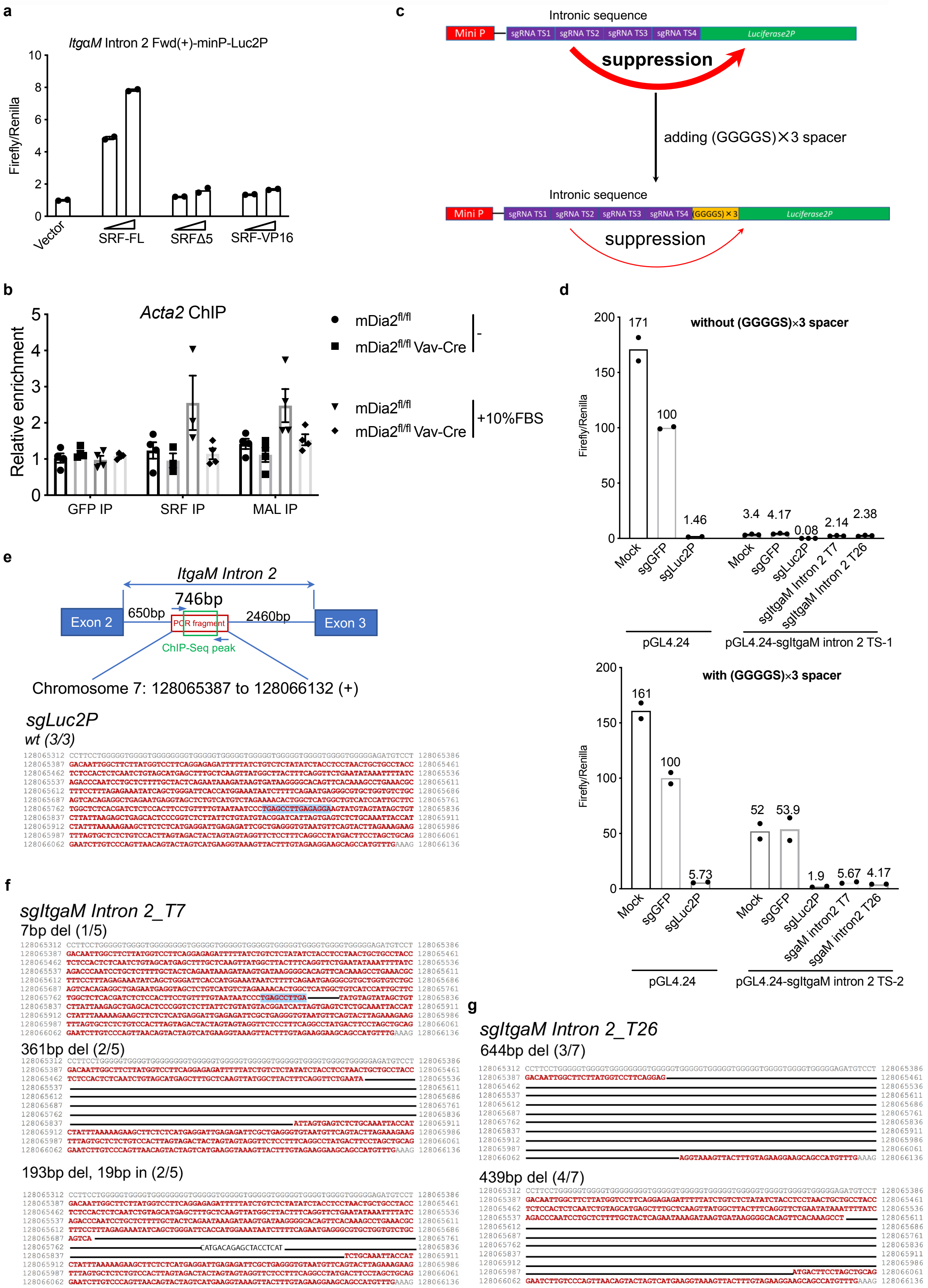

a
ItgαM Intron 2 Fwd(+)-minP-Luc2P
Firefly/Renilla
Vector
SRF-FL
SRFΔ5
SRF-VP16
b
Acta2 ChIP
Relative enrichment
GFP IP
SRF IP
MAL IP
mDia2fl/fl
mDia2fl/fl Vav-Cre
-
+10%FBS
c
Intronic sequence
Mini P
sgRNA TS1
sgRNA TS2
sgRNA TS3
sgRNA TS4
Luciferase2P
suppression
adding (GGGGS)×3 spacer
(GGGGS) × 3
d
without (GGGGS)×3 spacer
with (GGGGS)×3 spacer
Firefly/Renilla
171
100
1.46
3.4
4.17
0.08
2.14
2.38
161
100
5.73
52
53.9
1.9
5.67
4.17
Mock
sgGFP
sgLuc2P
sgItgaM Intron 2 T7
sgItgaM Intron 2 T26
sgaM intron2 T7
sgaM intron2 T26
pGL4.24
pGL4.24-sgItgaM intron 2 TS-1
pGL4.24-sgItgaM intron 2 TS-2
e
ItgaM Intron 2
746bp
650bp
2460bp
Exon 2
Exon 3
PCR fragment
ChIP-Seq peak
Chromosome 7: 128065387 to 128066132 (+)
sgLuc2P
wt (3/3)
f
sgItgaM Intron 2_T7
7bp del (1/5)
361bp del (2/5)
193bp del, 19bp in (2/5)
g
sgItgaM Intron 2_T26
644bp del (3/7)
439bp del (4/7)

Supplementary Figure 6. Targeting SRF intronic binding sites in *ItgaM* loucs via CRISPR-Cas9
(**a**) Luciferase activity assay with reporter construct containing *ItgaM* intronic regions co-transfected with increased amount of SRF full length (SRF-FL), exon 5 deficient isoform (SRFΔ5), or constitutively active SRF-VP16. Experiment was performed in duplicate. (**b**) Same ChIP assay was performed as in Fig. 4h except *Acta2* was analyzed by real-time PCR in quadruplicate. Error bars represent the SEM of the mean. (**c**) Schematic diagram showing the effect of (GGGGS)×3 spacer in luciferase reporter assay-based screening of sgRNA targeting of the intronic regions. The details were described in Fig. 6a and methods. (**d**) Quantitative analyses of the activities of recombinant luciferase harboring intronic DNA-encoded epitope without (upper) or with (lower) the (GGGGS)×3 spacer. sgLuc2P targeting luciferase gene was served as positive control. (GGGGS)×3 spacer largely reduced the inhibition of luciferase activity by intronic DNA encoded epitope. Experiments were performed in duplicate except the upper panel in triplicate. Repeated twice with similar results. (**e-g**) Determination of in vivo genome editing within *ItgaM* intron 2 region containing SRF binding element (CArG-like motif). The genomic DNA was isolated from the peripheral blood of the recipient mice and the target regions shown in the upper panel of e were PCR-amplified. The PCR products were cloned and subjected to sequencing to confirm the indels and estimate the editing frequencies. sgLuc2P, negative control, 100% (3/3) clones contain intact wide type sequence (lower panel in e); sgITGAM Intron 2_T7, 20% (1/5) clones contain a 7-bp nucleotide deletion occurred in the terminal region of CArG-like motif; 40% (2/5) clones contain a 361-bp deletion. 40% (2/5) clones contain a 193-bp deletion combined with a 19-bp insertion. They all lack the CArG-like motif (f); sgITGAM Intron 2_T26, 42.9% (3/7) clones contain a 644-bp deletion. 57.1% (4/7) clones contain a 439-bp deletion. Both lack the CArG-like motif (g).

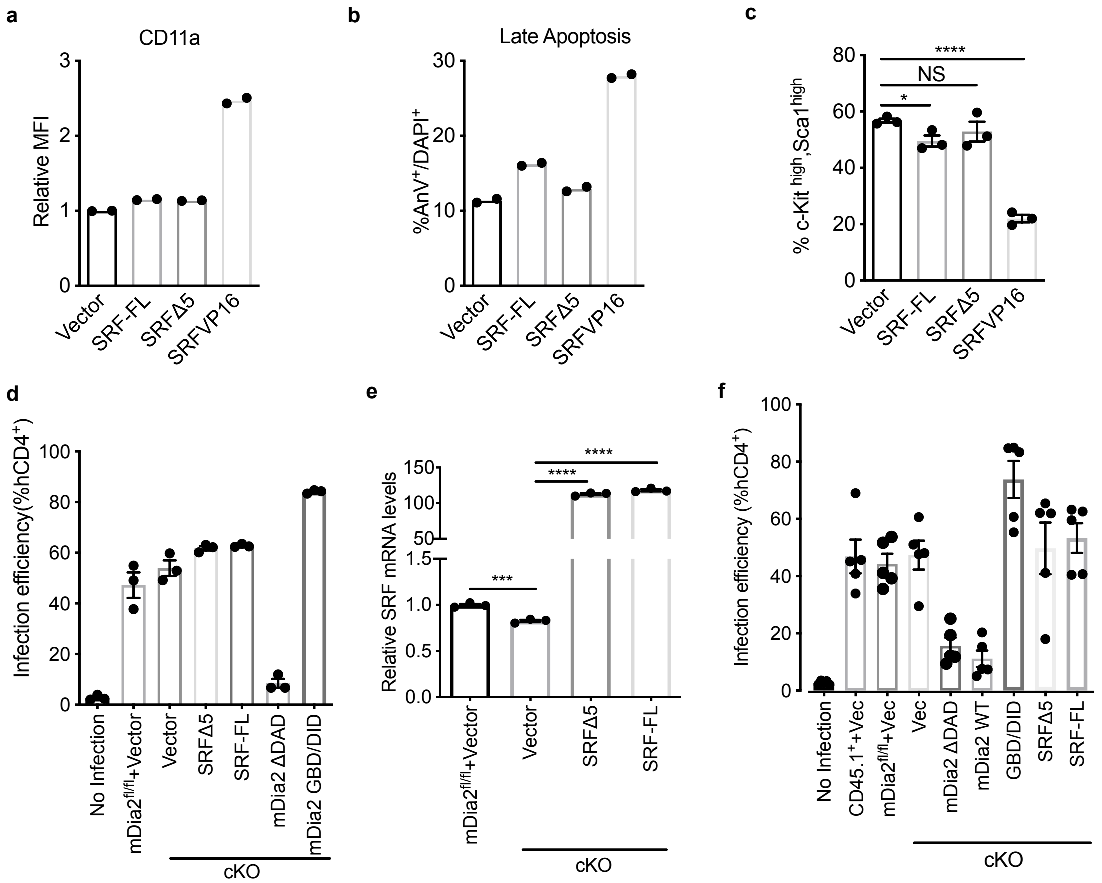
a
CD11a
Relative MFI
Vector
SRF-FL
SRFΔ5
SRFVP16
b
Late Apoptosis
%AnV+/DAPI+
c
% c-Kit high,Sca1high
****
NS
*
d
Infection efficiency(%hCD4+)
No Infection
mDia2fl/fl+Vector
mDia2 ΔDAD
mDia2 GBD/DID
cKO
e
Relative SRF mRNA levels
***
f
Infection efficiency (%hCD4+)
CD45.1+ +Vec
mDia2fl/fl+Vec
Vec
mDia2 WT
GBD/DID

Supplementary Figure 7. Expression of SRF and mDia2 variants (**a**) Ex vivo cultured c-Kit+ HSPCs transduced with indicated genes were examined for CD11a expression by flow cytometry**.** (**b**) Same as a except apoptosis was examined. *n*=2 mice in each group for a, b. (**c**) Stemness (c-kit^{high}/Sca1^{high}) was examined in ex vivo cultured c-Kit+ HSPCs. *n*=3 mice in each group. (**d**) Transduction efficiency of the indicated genes detected by flow cytometry of human CD4 co-expressed from the vector. *n*=3 mice in each group. (**e**) Quantitative analyses of the mRNA levels of SRF from indicated cells determined by quantitative RT-PCR. Experiment was performed in triplicate. (**f**) Transduction efficiency of the indicated constructs quantified by human CD4 expression through flow cytometry. *n*=5 mice in each group. Error bars represent the SEM of the mean. **p* < 0.05, ***p* < 0.01, ****p* < 0.001, *****p* < 0.0001. Two-tailed unpaired student's t test was used to generate the p values.

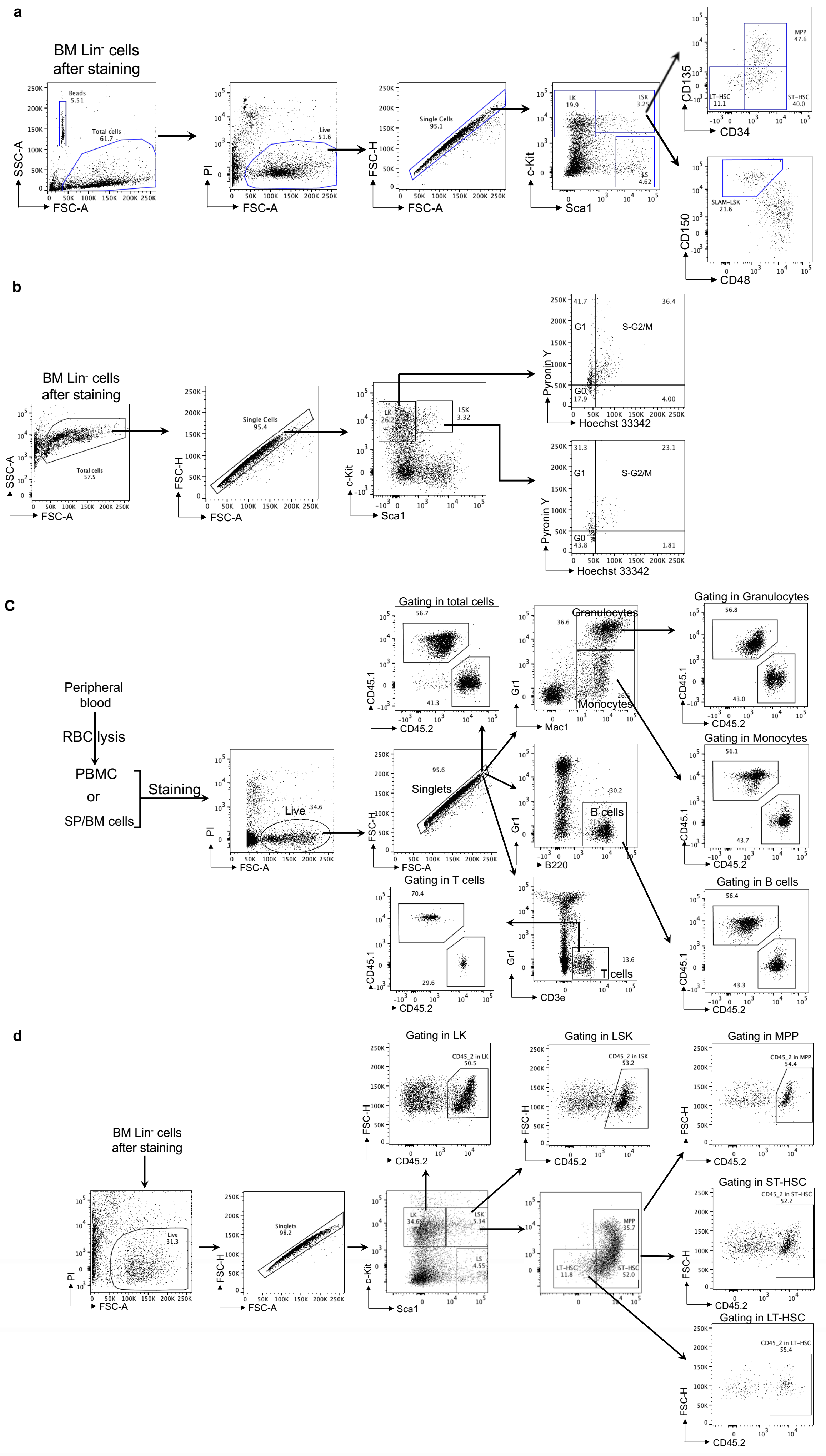
a
BM Lin⁻ cells after staining
Beads 5.51
Total cells 61.7
Live 51.6
Single Cells 95.1
LK 19.9
LSK 3.25
LS 4.62
MPP 47.6
LT-HSC 11.1
ST-HSC 40.0
SLAM-LSK 21.6
SSC-A
FSC-A
PI
FSC-H
c-Kit
Sca1
CD135
CD34
CD150
CD48
b
BM Lin⁻ cells after staining
Total cells 57.5
Single Cells 95.4
LK 26.2
LSK 3.32
41.7
36.4
G1
S-G2/M
G0 17.9
4.00
31.3
23.1
G0 43.8
1.81
Pyronin Y
Hoechst 33342
c
Peripheral blood
RBC lysis
PBMC
or
SP/BM cells
Staining
Live 34.6
Singlets 95.6
Gating in total cells
56.7
41.3
Granulocytes
36.6
Monocytes
26.
B cells
30.2
T cells
13.6
Gating in T cells
70.4
29.6
Gating in Granulocytes
56.8
43.0
Gating in Monocytes
56.1
43.7
Gating in B cells
56.4
43.3
CD45.1
CD45.2
Gr1
Mac1
B220
CD3e
d
BM Lin⁻ cells after staining
Live 31.3
Singlets 98.2
LK 34.6
LSK 5.34
LS 4.55
MPP 35.7
LT-HSC 11.8
ST-HSC 52.0
Gating in LK
CD45_2 in LK 50.5
Gating in LSK
CD45_2 in LSK 53.2
Gating in MPP
CD45_2 in MPP 54.4
Gating in ST-HSC
CD45_2 in ST-HSC 52.2
Gating in LT-HSC
CD45_2 in LT-HSC 55.4

Supplementary Figure 8. Flow cytometric gating strategies (**a**) Flow cytometric gating of LT-HSC,ST-HSC,MPP and SLAM-LSK for Fig. 1a, 5g and Supplementary Figure 1c-d, 2a, 2c-d. (**b**) Gating strategy for stem cell quiescence for Fig. 1d, 1e and Supplementary Figure 2b. (**c-d**) Gating routines for peripheral blood, bone marrow, spleen and HSPCs in competitive bone marrow transplantation as shown in Fig. 2b-c, 5e-g, 6h-i and Supplementary Figure 2h-i.

Supplementary Tables

Supplementary Table 1. Primer sequences for cloning, RT-PCR, qPCR and ChIP assays

Name	Sequence (5' - 3')	Purposes/Comments
ORF cloning into MICD4		
SRF-F	AAA GGATCC GCCGCC ATGTTACCGAGCCAAGCTGGGGCCGC	Forward and reverse primers for PCR amplification of mouse SRF ORF; BamHI and EcoRI restriction sites are highlighted in gray
SRF-R	GGG GAATTC TCATTCACTCTTGGTGCTGTGGGTGGC	
HA-SRF VP16-F	GGG GTTAAC GCCACC ATGGCTTCTAGCTATCCTTATG	Forward and reverse PCR primers containing HpaI and NotI restriction sites (gray).
HA-SRF VP16-R	AAA GCGGCCGC TTACTACCCACCGTAC	
Luciferase constructs cloning		
CD18-Intron 6-F	ACCTGAGCTCGCTAGCACATGGGTGC AGCAAGTG	Forward and reverse primers for 730bp *Itgβ2* intron 6 forward orientation cloning by In-Fusion. 15bp complementary overhangs are highlighted in gray.
CD18-Intron 6-R	TCTAGTGTCTAAGCTTAAAGTGGAAGC CATCGTCTGTG	
CD11b-Intron 2-F	ACCTGAGCTCGCTAGCGACAATTGGC TTCTTATGGTCC	Forward and reverse primers for 746bp *ItgαM* intron 2 forward orientation cloning by In-Fusion. 15bp complementary overhangs are highlighted in gray. sgItgaM Intron 2 target region PCR, TA-cloning and sequencing for indels verification.
CD11b-Intron 2-R	TCTAGTGTCTAAGCTTCAAACATGGCT GCTTCCTTC	
CD18 Intron 6-RCF	ACCTGAGCTCGCTAGTAAAGTGGAAG CCATCGTCTGTG	Forward and reverse primers for 730bp *Itgβ2* intron 6 reverse orientation cloning by In-Fusion. 15bp complementary overhangs are highlighted in gray.
CD18 Intron 6 RCR	TCTAGTGTCTAAGCTCACATGGGTGCA GCAAGTG	
CD11b Intron 2 RCF	ACCTGAGCTCGCTAGTCAAACATGGCT GCTTCCTTC	Forward and reverse primer for 746bp *ItgαM* intron 2 reverse orientation cloning by In-Fusion. 15bp complementary overhangs are highlighted in gray.
CD11b Intron 2 RCR	TCTAGTGTCTAAGCTCGACAATTGGCT TCTTATGGTCC	
Mutagenesis and deletion		
CD11b-AA mutant-F	TCCACTTCCTGTTTTGTAATAATCCCTG AGAATTGAGAAAAGTATGTAGTATAG CTGTCTTATTAAGAG	Forward and reverse primers for site-direct mutagenesis of *ItgαM* intron 2 SRE site. AA mutant in this study. Mutated nucleotides are highlighted in gray.
CD11b-AA mutant-R	CTCTTAATAAGACAGCTATACTACATAC TTTTTCTCAATTCTCAGGGATTATTACA AAACAGGAAGTGGA	
CD11b-TC mutant-F	CTGAGCCTTGAGAGGAAGTACGTAGT ATAGCTGTCTTATTA	Forward and reverse primers for site-direct mutagenesis of *ItgαM* intron 2 SRE site. TC mutant in this study. Mutated nucleotides are highlighted in gray.
CD11b-TC mutant-R	TAATAAGACAGCTATACTACGTACTTC CTCTCAAGGCTCAG	
CD11b-AATC mutant-F	CCCTGAGAATTGAGAAAAAGTACGTAG TATAGCTGTCTTATTAAGAGCTGAGCA CTC	Forward and reverse primers for site-direct mutagenesis of *ItgαM* intron 2 SRE site. AATC mutant in this study. Mutated nucleotides are highlighted in gray.
CD11b-AATC mutant-R	GCTATACTACGTACTTTTTCTCAATTCT CAGGGATTATTACAAAACAGGAAGTGG AGAGATC	
CD18-MF	CTTCTTTTGTGGCTGGGAGGTGTTTGC ATTTTAAAATACAAAAAAATACTCTTTT CCTTTCATGACTGTGTTG	Forward and reverse primers for site-direct mutagenesis of *Itgβ2* intron 6 SRE site in luciferase construct. Mutated nucleotides are highlighted in gray.
CD18-MR	CAACACAGTCATGAAAGGAAAAGAGTA TTTTTTTGTATTTTAAAATGCAAACACC TCCCAGCCACAAAAGAAG	

CD18-Deletion-F	TGGCTGGGAGGTGTTTAATCTAGTTTC AGGTCTGAAGAGGGTTGCAAAGACATT TCTC	Forward and reverse primers for 52bp deletion of ItgαM intron 2 region harboring SRE site.
CD18- Deletion-R	GACCTGAAACTAGATTAAACACCTCCC AGCCACAAAAGAAGGGACCAAGGGAC ATCCA	
Quantitative PCR		
Acta2	F: GGCACCACTGAACCCTAAGG<br>R: ACAATACCAGTTGTACGTCCAGA	qRT-PCR of *Acta2*, in Exon 4 and 5/6[3].
Krt17	F: AGCCGCATCCTGTCAGAGAT<br>R: CGGCCATCACGCCACAGTTT	qRT-PCR of *KRT-17*, both in Exon 4[4].
Flna	F: GAGTTCGGCATTTGGACTAGG<br>R: GGGCTATCAGGTATGTGCTCC	qRT-PCR of *Flna*, in Exon 41and 42[3].
Fhl2	F: GATCGGCACTGGCATGAAG<br>R: AGCAAAGGGCTTGTCCACC	qRT-PCR of *FHL2*, both in Exon 3[4].
Myh9	F: GGCCCTGCTAGATGAGGAGT<br>R: CTTGGGCTTCTGGAACTTGG	qRT-PCR of *Myh9*, both in Exon 14[3].
Actb	F: GGCTGTATTCCCCTCCATCG<br>R: CCAGTTGGTAACAATGCCATGT	qRT-PCR of *Actb*, in Exon 2 and 3[3].
Actg1	F: CGTCCACCGCAAATGCTTC<br>R: TGCCAGGGCAAATTGATACT	qRT-PCR of *Actg1*, both in Exon 6[5].
Srf	F: GCAAGGCGCTGATTCAGAC<br>R: TCAGATTCCGACACCTGGTAG	qRT-PCR of *Srf*, both in Exon 2[3].
Egr3	F: AGCCCAATCCGGAACTCTCTT<br>R: GGAAGGAGAGTCGAAAGCGAA	qRT-PCR of *Egr3*, both in Exon 2[6].
Egr1	F: ATTGATGTCTCCGCTGCAGATC<br>R: TCAGCAGCATCATCTCCTCCA	qRT-PCR of *Egr1*, both in Exon 1[6].
Fosb	F: GAGGGAGCTGACAGATCGAC<br>R: AAACTCCAGGCGTTCCTTCT	qRT-PCR of *Fosb*, designed by Primer3 (version 0.4.0), in Exon 3 and 4.
c-Fos	F: TGACTGGAGGTCTGCCTGAGGCTT<br>R: GCTCCAAGGATGGCTTGGGCTC	qRT-PCR of Egr1, both in Exon 4[7].
Coro1c	F: CGCAGAGCGTGCTTATTCG<br>R: TGCCAACCATTTCCAAAACTAA	qRT-PCR of *Coro1c* intron-containing pre-mRNA, both in Intron 1[7].
Jun B	F: ACAAGGTGAAGACACTCAAGGCT<br>R: ATGACCTTCTGCTTGAGCTGC	qRT-PCR of *JunB*, both in Exon 1[6].
Egr2	F: GAGCAAATGATGACCGCCAA<br>R: TGTCAGGCAGCTGGTGCATAA	qRT-PCR of *Egr2*, both in Exon 1[6].
Vcl	F: AGCCCAGATGCTTCAGTCAGA<br>R: GGTCAGATGTGCCAGAAAGGA	qRT-PCR of *Vcl*, both in Exon 3[3]
Nr4a1	F: GCTTGGCACCCACCTCTCCGA<br>R: CACGGGTGCGTCCAGAATGCC	qRT-PCR of *Nr4a1*, both in Exon 2[7].
Tuftelin	F: AGGAAGTGGCTGGGTTTCG<br>R: GAACTTTCCGCTGCTGGCT	qRT-PCR of *Tuftelin*, both in Exon 10[4].
Tagln	F: ACCAAAAACGATGGAAACTACCG<br>R: GTGAAGTCCCTCTTATGCTCCT	qRT-PCR of *Tagln*, both in Exon 4-5[3].
Itga1	F: CAGTGGAGGACATGTTTGGAT<br>R: TCTCTCTCTCCCAACTGGACA	qRT-PCR of *Itga1*, designed by Primer3 (version 0.4.0), in Exon 2 and 3.
Itga2	F: GCAACTGGCTACTGGTTGGT<br>R: AGCTTTTCACAGGTGGCAGT	qRT-PCR of *Itga2*, designed by Primer3 (version 0.4.0), in Exon 2/3 and 3.
Itga4	F: GGGCTTGTGAACCCAACTTC<br>R: TGCATGTTTCTGGCTCGTTTT	qRT-PCR of *Itga4*, both in Exon 22[5].
Itga5	F: TTCTCCGTGGAGTTTTACCG<br>R: TAGACAGCACCACCTTGCAG	qRT-PCR of *Itga5*, designed by Primer3 (version 0.4.0), in Exon 1 and 2.
ItgaL	F: CCCGCTTGGTCGGTTTG<br>R: CAGTCAGCCTATCCCCATTGA	qRT-PCR of *ItgaL*, both in Exon 14[5].
ItgaM	F: CTGAACATCCCATGACCTTCC<br>R: GCCCAAGGACATATTCACAGC	qRT-PCR of *ItgaM*, in Exon 2 and 4[8].

Itgb1	F: GGTTTCCTGGATTGGATTGA<br>R: CAATTTGGCCCTGCTTGTAT	qRT-PCR of *Itgb1*, designed by Primer3 (version 0.4.0), in Exon 2 and 3.
Itgb2	F: TTTCGGCACGTGCTCAAG<br>R: TTGCCGACCTCTGTCTGAAAC	qRT-PCR of *Itgb2*, both in Exon 6[5].
RT-PCR		
SRF RT-PCR-F	CCTCAACTCGCCAGACTCTC	Forward and reverse primers for RT-PCR detecting murine SRF truncations.
SRF RT-PCR-R	CAGGAACACCTGAGGGACAC	
gBlock DNA		
Itgb2/aM CDS	ggtaaagccaccatgaAGTACAAAGTCAGCAGTTGCCGGGACTGTATCCAGTCGGGGCCTGGCTGTTCCTGGTGCCAGAAGCTGTCACTGCTGCTTGCCCTAGCTGGACTGTTCTTCCTGGGATCTGcACGGGCACAGCTGCTGCTGAAGGGTTGTCCAGCCGATGATATCATGGACCCCAGGAGCATCGCTAATCCTGAGTTCGACCAACGGGGGCAACGGAAACAGCCCCATGACCTTCCAAGAGAATGCAAAAGGCTTTGGACAGAGTGTGGTCCAGCTTGGCGGACCAGTGTGGTTGTTGCAGCCCCCCAGGAGGCAAAGGCTGTTAACCAGACAGGtgaagatgccaaaaacattaagaagggcccagcgccattctacc	Oligo sequence containing potential sgRNA target sites for both *Itgb2* and *ItgaM* protein coding regions was shown in uppercase.
ItgaM intron 2	ggtaaagccaccatgATCCGTACATACAGAATAAGAGACCGGGGCTATACTACATACTTCCTCTCAAGGCTCAGGGATTATTACAACAGCCTCTGTGACTGCAGACACCAGCACGGTGGAGGTGGAAGTGGAGGTGGAGGTTCTGGAGGCGGGGGTAGCgaagatgccaaaaacattaagaagggcccagcgccattctacc	Oligo sequence containing potential sgRNA target sites covering SRE in *ItgaM* intron 2 region was shown in uppercase, and underlined sequence encoded (GSSSS)×3 spacer.
15bp-F	ggtaaagccaccatg	PCR primers for gBlock amplification to introduce 15bp overhang for In-Fusion reactions.
15bp-R	ggtagaatggcgctg	
sgRNA sequence		
sgLuc2P		
sgItgb2_ T6	F: CACCg ATCCTGAGTTCGACCAACGG<br>R: AAAC CCGTTGGTCGAACTCAGGATC	sgRNA targeting Itgb2 Exon4 with high efficiency, used in all subsequent experiments.
sgItgb2_ T13	F: CACCg AGTCGGGGCCTGGCTGTTCC<br>R: AAAC GGAACAGCCAGGCCCCGACTC	sgRNA targeting Itgb2 Exon3 with minimal suppression effect.
sgItgaM_ T4	F: CACCg CAGAGTGTGGTCCAGCTTGG<br>R: AAAC CCAAGCTGGACCACACTCTGC	sgRNA targeting ItgaM Exon2 with high efficiency, used in all subsequent experiments.
sgItgaM_ T12	F: CACCg TAACAGCCTTTGCCTCCTGG<br>R: AAAC CCAGGAGGCAAAGGCTGTTAC	sgRNA targeting ItgaM Exon3 with lower efficiency.
sgItgaM_ intron 2_T7	F: CACCgATACTACATACTTCCTCTCA<br>R: AAACTGAGAGGAAGTATGTAGTATC	sgRNA targeting ItgaM intron 2 SRF binding site.
sgItgaM_intron 2_T26	F: CACCgATACTTCCTCTCAAGGCTCA<br>R: AAACTGAGCCTTGAGAGGAAGTATC	sgRNA targeting ItgaM intron 2 SRF binding site.
ChIP qRT-PCR primer sequence		
ChIP-ItgaM	F: GGCTGTCATCCATTGCTTCT<br>R: ACCCTCAGCGAATCTCTCAA	qRT-PCR primers for ChIP assay detecting SRF binding in ItgaM

ChIP-Itgb2	F: CTGGGAGGTGTTTGCATTTT<br>R: CAATCCAGTGCCAAGGAAGT	qRT-PCR primers for ChIP assay detecting SRF binding in Itgb2
ChIP-LINE1	F: AAACGAGGAGTTGGTTCTTTGAG<br>R: TTTGTCCCTGTGCCCTTTAGTGA	qRT-PCR primers of negative control for ChIP assay
ChIP-Acta2	F: GAGGCCTGGGTCTCTTCCA<br>R: GCTGAGCTGCCTCCTGTTTC	qRT-PCR primers for ChIP assay detecting SRF/MAL binding in Acta2

Supplementary Table 2. Antibodies used for FACS and immunofluorescence staining

Antibodies for flow cytometric assay		
Antibody	Catalog number	Supplier or comments
APC-CD11b(human)	ICRF44, Cat# 301310	BioLegend, 1:100 dilution
APC-CD11b(mouse)	M1/70, Cat# 17-0112-82	eBioscience, 1:100 dilution
APC-CD18	C71/16, Cat# 562828	BD Pharmingen, 1:100 dilution
APC-CD11a	M17/4, Cat# 101119	BioLegend, 1:100 dilution
BV421-CD18	M18/2, Cat# 744597	BD OptiBuild, 1:100 dilution
V500-CD11b	M1/70, Cat# 562127	BD Horizon, 1:100 dilution
Biotin-c-Kit	2B8, Cat# 13-1171-85	eBioscience, 1:100 dilution
PE-CD11b	M1/70, Cat# 553311	BD Pharmingen, 1:100 dilution
PE-Cy7-Gr1	RB6-8C5, Cat# 108416	BioLegend, 1:100 dilution
Pacific Blue-B220	RA3-6B2, Cat# 103227	BioLegend, 1:100 dilution
APC-eFluor780-CD3e	17A2, Cat# 47-0032-82	eBioscience, 1:100 dilution
APC/Fire 750-CD3e	17A2, Cat# 100248	BioLegend, 1:100 dilution
FITC-CD45.2	104, Cat# 109806	BioLegend, 1:100 dilution
V500-CD45.2	104, Cat# 562130	BD Horizon, 1:100 dilution
APC-CD45.1	A20, Cat# 110714	BioLegend, 1:100 dilution
PE-Sca1(Ly-6A/E)	D7, Cat# 108108	BioLegend, 1:100 dilution
PE-Cy7-CD117(c-Kit)	2B8, Cat# 105814	BioLegend, 1:100 dilution
APC-CD135	A2F10, Cat# 135310	BioLegend, 1:100 dilution
BV421-CD34	RAM34, Cat# 562608	BD Horizon, 1:100 dilution
PerCP-Cy5.5-CD16/CD32	93, Cat# 101323	BioLegend, 1:100 dilution
Pacific Blue-CD117 (c-Kit)	2B8, Cat# 105820	BioLegend, 1:100 dilution
APC-CD150 (SLAM)	TC15-12F12.2, Cat# 115910	BioLegend, 1:100 dilution
APC-eFlour780-CD48	HM48-1, Cat# 47-0481-82	eBioscience, 1:100 dilution
PerCP-Cy5.5-CD127(IL-7Ra)	A7R34, Cat# 135022	BioLegend, 1:100 dilution
APC-CD184 (CXCR4)	2B11, Cat# 17-9991-80	eBioscience, 1:100 dilution
APC-CD117(c-Kit)	2B8, Cat# 17-1171-82	eBioscience, 1:100 dilution
Antibodies for immunofluorescence staining		
Alexa Fluor 594 Phalloidin	Cat# A12381	Thermo Fisher Scientific, 165 nM
Alexa Fluor 647 anti-Tubulin-α	Cat# 627908	BioLegend, 1:300 dilution
Anti-MAL	Cat# sc-390324	Santa Cruz Biotechnology, 1:200 dilution
anti-Lamin B1	Cat# ab16048	Abcam, 1:200 dilution
Alex Fluor 594 goat anti-mouse IgG	Cat# A-11005	Thermo Fisher Scientific, 1:400 dilution
Alex Fluor 647 donkey anti-rabbit IgG	Cat# A-31573	Thermo Fisher Scientific, 1:400 dilution
Antibodies for other use		
PE-CD31	Cat# 12-0311-82	Thermo Fisher Scientific, 3μg/ml
anti-SRF	2C5, Cat# 61386	Active Motif, 3-5μg per ChIP
anti-MAL	Cat# sc-390324	Santa Cruz Biotechnology,3-5μg per ChIP
anti-GFP	B2, Cat# sc-9996	Santa Cruz Biotechnology,3-5μg per ChIP

Supplementary References

1. Seita, J. *et al.* Gene Expression Commons: an open platform for absolute gene expression profiling. *PLoS One* **7**, e40321 (2012).
2. Sun, D. *et al.* Epigenomic profiling of young and aged HSCs reveals concerted changes during aging that reinforce self-renewal. *Cell Stem Cell* **14**, 673-688 (2014).
3. Vasudevan, H.N. & Soriano, P. SRF regulates craniofacial development through selective recruitment of MRTF cofactors by PDGF signaling. *Dev Cell* **31**, 332-344 (2014).
4. Philippar, U. *et al.* The SRF target gene Fhl2 antagonizes RhoA/MAL-dependent activation of SRF. *Mol Cell* **16**, 867-880 (2004).
5. Costello, P. *et al.* MRTF-SRF signaling is required for seeding of HSC/Ps in bone marrow during development. *Blood* **125**, 1244-1255 (2015).
6. Costello, P. *et al.* Ternary complex factors SAP-1 and Elk-1, but not net, are functionally equivalent in thymocyte development. *J Immunol* **185**, 1082-1092 (2010).
7. Mylona, A. *et al.* The essential function for serum response factor in T-cell development reflects its specific coupling to extracellular signal-regulated kinase signaling. *Mol Cell Biol* **31**, 267-276 (2011).
8. Mei, Y. *et al.* Loss of mDia1 causes neutropenia via attenuated CD11b endocytosis and increased neutrophil adhesion to the endothelium. *Blood Adv* **1**, 1650-1656 (2017).
