# Supplementary material for: Diaphanous-related formin mDia2 regulates beta2 integrins to control hematopoietic stem and progenitor cell engraftment
Source: Nat Commun. 2020 Jun 23;11:3172. doi: 10.1038/s41467-020-16911-4 (PMC7311390; doi:10.1038/s41467-020-16911-4)
Supplement: Supplementary file 3 — Reporting Summary [file 41467_2020_16911_MOESM3_ESM.pdf]

nature research

Corresponding author(s): Peng Ji/Yang Mei

Last updated by author(s): May 22, 2020

Reporting Summary

Nature Research wishes to improve the reproducibility of the work that we publish. This form provides structure for consistency and transparency in reporting. For further information on Nature Research policies, seeAuthors & Referees and theEditorial Policy Checklist .

Statistics

For all statistical analyses, confirm that the following items are present in the figure legend, table legend, main text, or Methods section.

n/a	Confirmed	
☐	☒	The exact sample size (*n*) for each experimental group/condition, given as a discrete number and unit of measurement
☐	☒	A statement on whether measurements were taken from distinct samples or whether the same sample was measured repeatedly
☐	☒	The statistical test(s) used AND whether they are one- or two-sided<br>*Only common tests should be described solely by name; describe more complex techniques in the Methods section.*
☒	☐	A description of all covariates tested
☒	☐	A description of any assumptions or corrections, such as tests of normality and adjustment for multiple comparisons
☐	☒	A full description of the statistical parameters including central tendency (e.g. means) or other basic estimates (e.g. regression coefficient) AND variation (e.g. standard deviation) or associated estimates of uncertainty (e.g. confidence intervals)
☐	☒	For null hypothesis testing, the test statistic (e.g. *F*, *t*, *r*) with confidence intervals, effect sizes, degrees of freedom and *P* value noted<br>*Give P values as exact values whenever suitable.*
☒	☐	For Bayesian analysis, information on the choice of priors and Markov chain Monte Carlo settings
☒	☐	For hierarchical and complex designs, identification of the appropriate level for tests and full reporting of outcomes
☒	☐	Estimates of effect sizes (e.g. Cohen's *d*, Pearson's *r*), indicating how they were calculated

Our web collection on statistics for biologists contains articles on many of the points above.

Software and code

Policy information about availability of computer code

Data collection	No software was used for data collection.
Data analysis	Statistical comparisons between two groups were performed with a two-tailed unpaired Student's t test using GraphPad Prism software (Version 8.0). Survival curves were compiled using Kaplan-Meier algorithms in Prism software, and the significance was assessed using Mantel-Cox log-rank test. The real-time PCR data were analyzed and exported by StepOnePlus software(v2.3), NIS Elements software and Volocity® software (Perkin Elmer,v6.1) were applied for immunofluorescence staining imaging and in vivo imaging process respectively. The images were further analyzed by Image J (1.51m9).

For manuscripts utilizing custom algorithms or software that are central to the research but not yet described in published literature, software must be made available to editors/reviewers. We strongly encourage code deposition in a community repository (e.g. GitHub). See the Nature Research guidelines for submitting code & software for further information.

Data

Policy information about availability of data

All manuscripts must include a data availability statement. This statement should provide the following information, where applicable:

- Accession codes, unique identifiers, or web links for publicly available datasets
- A list of figures that have associated raw data
- A description of any restrictions on data availability

The data that support the findings of this study have been uploaded online as source data file. The Gene Expression Commons database was open-accessed upon registration (https://gexc.riken.jp). The DNA sequences from ChIP-seq peak were recalled by UCSC genome browser (MM8), and the location of the binding peaks were further illustrated within the genomic locus from open-accessed Ensemble database in Figure 4A. The gene expression and associated epigenetic information in HSC as shown in supplementary Figure 5D-E were extracted from the public HSC Aging Hub (http://dldcc-web.brc.bcm.edu/lilab/AgingHSCEpigenome/browser.html). Source data underlying Fig. 1b-c,e, 2c-d, f-g, i, k, 3c-e, h, j-l, 4c-j, 5a, c, e-g, 6b-f, h-i and Supplementary Figure 1a, d, f, 2a-d, g, i, 3a-c, f, 4b-d, 5b–c, 6a-b, d, and 7a-f are available as a Source Data file. Other data that support the findings of this study are available from the corresponding author upon request.

Field-specific reporting

Please select the one below that is the best fit for your research. If you are not sure, read the appropriate sections before making your selection.

- [x] Life sciences
- [ ] Behavioural & social sciences
- [ ] Ecological, evolutionary & environmental sciences

For a reference copy of the document with all sections, see nature.com/documents/nr-reporting-summary-flat.pdf

Life sciences study design

All studies must disclose on these points even when the disclosure is negative.

Sample size	Experiments were usually performed with 3 or more biological repeats. For Figure 2g, 3h-j, 4i-j, 6e, n=2 from two independent experiments. For Figure 6i, data were collected from n=2 mice.
Data exclusions	No data were excluded from the analysis except for the failed amplification wells in real-time PCR.
Replication	Experiments were successfully repeated at least twice. Multiple samples, animals and clones were included.
Randomization	Animals were assigned to groups depending on their genotypes. During flow data collection, the samples were deidentified, and were identified when analyzing data.
Blinding	The investigators were not blinded to allocate the groups during experiments.

Reporting for specific materials, systems and methods

We require information from authors about some types of materials, experimental systems and methods used in many studies. Here, indicate whether each material, system or method listed is relevant to your study. If you are not sure if a list item applies to your research, read the appropriate section before selecting a response.

Materials & experimental systems

n/a	Involved in the study	
[ ]	[x]	Antibodies
[ ]	[x]	Eukaryotic cell lines
[x]	[ ]	Palaeontology
[ ]	[x]	Animals and other organisms
[x]	[ ]	Human research participants
[x]	[ ]	Clinical data

Methods

n/a	Involved in the study	
[x]	[ ]	ChIP-seq
[ ]	[x]	Flow cytometry
[x]	[ ]	MRI-based neuroimaging

Antibodies

Antibodies used

Antibodies for flow cytometric assay:
APC-CD11b(human) ICRF44, Cat# 301310 BioLegend, 1:100 dilution
https://www.biolegend.com/en-us/products/apc-anti-human-cd11b-antibody-765

APC-CD11b(mouse) M1/70, Cat# 17-0112-82 eBioscience, 1:100 dilution
https://www.thermofisher.com/cn/en/antibody/product/CD11b-Antibody-clone-M1-70-Monoclonal/17-0112-82

APC-CD18 C71/16, Cat# 562828 BD Pharmingen, 1:100 dilution
https://www.bdbiosciences.com/us/applications/research/stem-cell-research/mesoderm-markers/mouse/apc-rat-anti-mouse-cd18-c7116/p/562828

APC-CD11a M17/4, Cat# 101119 BioLegend, 1:100 dilution
https://www.biolegend.com/en-us/products/apc-anti-mouse-cd11a-antibody-9069

BV421-CD18 M18/2, Cat# 744597 BD OptiBuild, 1:100 dilution
https://www.bdbiosciences.com/us/reagents/research/antibodies-buffers/immunology-reagents/anti-mouse-antibodies/cell-surface-antigens/bv421-rat-anti-mouse-cd18-m182/p/744597

V500-CD11b M1/70, Cat# 562127 BD Horizon, 1:100 dilution
https://www.bdbiosciences.com/us/applications/research/stem-cell-research/mesenchymal-stem-cell-markers-bone-marrow/mouse/negative-markers/v500-rat-anti-cd11b-m170/p/562127

Biotin-c-Kit 2B8, Cat# 13-1171-85 eBioscience, 1:100 dilution
https://www.thermofisher.com/cn/en/antibody/product/CD117-c-Kit-Antibody-clone-2B8-Monoclonal/13-1171-82

PE-CD11b M1/70, Cat# 553311 BD Pharmingen, 1:100 dilution
https://www.bdbiosciences.com/us/applications/research/stem-cell-research/mesenchymal-stem-cell-markers-bone-marrow/mouse/negative-markers/pe-rat-anti-cd11b-m170/p/553311

PE-Cy7-Gr1 RB6-8C5, Cat# 108416 BioLegend, 1:100 dilution
https://www.biolegend.com/en-gb/products/pe-cyanine7-anti-mouse-ly-6g-ly-6c-gr-1-antibody-1931

Pacific Blue-B220 RA3-6B2, Cat# 103227 BioLegend, 1:100 dilution
https://www.biolegend.com/en-gb/products/pacific-blue-anti-mouse-human-cd45r-b220-antibody-2857

APC-eFluor780-CD3e 17A2, Cat# 47-0032-82 eBioscience, 1:100 dilution
https://www.thermofisher.com/cn/en/antibody/product/CD3-Antibody-clone-17A2-Monoclonal/47-0032-82

APC/Fire 750-CD3e 17A2, Cat# 100248 BioLegend, 1:100 dilution
https://www.biolegend.com/en-gb/products/apc-fire-750-anti-mouse-cd3-antibody-13052

FITC-CD45.2 104, Cat# 109806 BioLegend, 1:100 dilution
https://www.biolegend.com/en-gb/products/fitc-anti-mouse-cd45-2-antibody-6

V500-CD45.2 104, Cat# 562130 BD Horizon, 1:100 dilution
https://www.bdbiosciences.com/us/applications/research/stem-cell-research/cancer-research/mouse/v500-mouse-anti-mouse-cd452-104/p/562130

APC-CD45.1 A20, Cat# 110714 BioLegend, 1:100 dilution
https://www.biolegend.com/en-gb/products/apc-anti-mouse-cd45-1-antibody-2319

PE-Sca1(Ly-6A/E) D7, Cat# 108108 BioLegend, 1:100 dilution
https://www.biolegend.com/en-gb/products/pe-anti-mouse-ly-6a-e-sca-1-antibody-228

PE-Cy7-CD117(c-Kit) 2B8, Cat# 105814 BioLegend, 1:100 dilution
https://www.biolegend.com/en-gb/products/pe-cyanine7-anti-mouse-cd117-c-kit-antibody-1900

APC-CD135 A2F10, Cat# 135310 BioLegend, 1:100 dilution
https://www.biolegend.com/en-gb/products/apc-anti-mouse-cd135-antibody-6284

BV421-CD34 RAM34, Cat# 562608 BD Horizon, 1:100 dilution
https://www.bdbiosciences.com/us/applications/research/stem-cell-research/cancer-research/mouse/bv421-rat-anti-mouse-cd34-ram34/p/562608

PerCP-Cy5.5-CD16/CD32 93, Cat# 101323 BioLegend, 1:100 dilution
https://www.biolegend.com/en-gb/products/percp-cyanine5-5-anti-mouse-cd16-32-antibody-6165

Pacific Blue-CD117 (c-Kit) 2B8, Cat# 105820 BioLegend, 1:100 dilution
https://www.biolegend.com/en-gb/products/pacific-blue-anti-mouse-cd117-c-kit-antibody-3133

APC-CD150 (SLAM) TC15-12F12.2, Cat# 115910 BioLegend, 1:100 dilution
https://www.biolegend.com/en-gb/products/apc-anti-mouse-cd150-slam-antibody-2894

APC-eFlour780-CD48 HM48-1, Cat# 47-0481-82 eBioscience, 1:100 dilution
https://www.thermofisher.com/cn/en/antibody/product/CD48-Antibody-clone-HM48-1-Monoclonal/47-0481-82

PerCP-Cy5.5-CD127(IL-7Ra) A7R34, Cat# 135022 BioLegend, 1:100 dilution
https://www.biolegend.com/en-gb/products/percp-cyanine5-5-anti-mouse-cd127-il-7ralpha-antibody-6196

APC-CD184 (CXCR4) 2B11, Cat# 17-9991-80 eBioscience, 1:100 dilution
https://www.thermofisher.com/cn/en/antibody/product/CD184-CXCR4-Antibody-clone-2B11-Monoclonal/17-9991-82

APC-CD117(c-Kit) 2B8, Cat# 17-1171-82 eBioscience, 1:100 dilution
https://www.thermofisher.com/cn/en/antibody/product/CD117-c-Kit-Antibody-clone-2B8-Monoclonal/17-1171-82

Antibodies for immunofluorescence staining:

Alexa Fluor 594 Phalloidin Cat# A12381 Thermo Fisher Scientific, 165 nM
https://www.thermofisher.com/order/catalog/product/A12381

Alexa Fluor 647 anti-Tubulin-α Cat# 627908 BioLegend, 1:300 dilution
https://www.biolegend.com/en-gb/products/alexa-fluor-647-anti-tubulin-alpha-antibody-9562

Anti-MAL Cat# sc-390324 Santa Cruz Biotechnology, 1:200 dilution
Validated in publication: PMID: # 32001718 Nat Commun. 11: 605.

anti-Lamin B1 Cat# ab16048 Abcam, 1:200 dilution
https://www.abcam.com/lamin-b1-antibody-nuclear-envelope-marker-ab16048.html

Alex Fluor 594 goat anti-mouse IgG Cat# A-11005 Thermo Fisher Scientific, 1:400 dilution
https://www.thermofisher.com/cn/en/antibody/product/Goat-anti-Mouse-IgG-H-L-Cross-Adsorbed-Secondary-Antibody-Polyclonal/A-11005

Alex Fluor 647 donkey anti-rabbit IgG Cat# A-31573 Thermo Fisher Scientific, 1:400 dilution
https://www.thermofisher.com/cn/en/antibody/product/Donkey-anti-Rabbit-IgG-H-L-Highly-Cross-Adsorbed-Secondary-Antibody-Polyclonal/A-31573

Antibodies for other use:
PE-CD31 Cat# 12-0311-82 Thermo Fisher Scientific, 3μg/ml
https://www.thermofisher.com/cn/en/antibody/product/CD31-PECAM-1-Antibody-clone-390-Monoclonal/12-0311-82

anti-SRF 2C5, Cat# 61386 Active Motif, 3-5μg per ChIP
https://www.activemotif.com/catalog/details/61385/srf-antibody-mab-clone-2c5
Validated by the vendor for ChIP-Sequencing.

anti-MAL Cat# sc-390324 Santa Cruz Biotechnology,3-5μg per ChIP

anti-GFP B2, Cat# sc-9996 Santa Cruz Biotechnology,3-5μg per ChIP
https://www.scbt.com/p/gfp-antibody-b-2

Validation

All antibodies used in this work are commercially available and have been validated in previous publications as well as in the vendors' websites. The website links are provided above with relevant publications.

Eukaryotic cell lines

Policy information about cell lines

Cell line source(s)

Lenti-X 293T(TaKaRa); Mouse endothelial cells(Cell Biologics)

Authentication

Cell lines from TakaRa and Cell Biologics were authenticated by the vendors themselves.

Mycoplasma contamination

Mycoplasma contamination was not checked in the study.

Commonly misidentified lines
(See ICLAC register)

No commonly misidentified cell lines were used in the study.

Animals and other organisms

Policy information about studies involving animals; ARRIVE guidelines recommended for reporting animal research

Laboratory animals

Congenic mice carrying CD45.1 antigen were purchased from Charles River (B6-LY-5.2/Cr, strain code: 564). C57/BL6 wild-type mice, CAG-Cas9 transgenic mice (stock #026179), Mx-Cre mice (stock #003556) and Vav-Cre mice (stock #008610) were purchased from the Jackson Laboratory. Both male and female mice were used in this study.

Mice were housed with a maximum of 5 in each cage at room temperature (25 °C) with a 12/12 hours light-dark cycle in the animal holding facilities at Northwestern University. The mice were supplied with a house and nesting material with free access to water and chow diet.

Wild animals

No wild animals were involved in this study.

Field-collected samples

No field collected samples were used in the study.

Ethics oversight

All the experiments involving animals were conducted in accordance with the Guide for the Care and Use of Laboratory Animals and were approved by the Institutional Animal Care and Use Committee at Northwestern University.

Note that full information on the approval of the study protocol must also be provided in the manuscript.

Flow Cytometry

Plots

Confirm that:

- [x] The axis labels state the marker and fluorochrome used (e.g. CD4-FITC).
- [x] The axis scales are clearly visible. Include numbers along axes only for bottom left plot of group (a 'group' is an analysis of identical markers).
- [x] All plots are contour plots with outliers or pseudocolor plots.
- [x] A numerical value for number of cells or percentage (with statistics) is provided.

Methodology

Sample preparation	To prepare cell suspensions from peripheral blood, 5-8 drops of tail blood were collected directly into 1 ml FACS buffer (1× PBS containing 0.5% BSA and 2 mM EDTA). After centrifugation at 8,000 rpm for 5 min, the cell pellets were lysed by incubation in 1× RBC lysis buffer (Catalog # 00-4333-57, Thermo Fisher Scientific) for 5 min. The nucleated cells (white blood cells) were recovered and washed by centrifugation at 8,000 rpm for 5 min before the cells were stained with specific antibodies for the analyses of lineage distributions.<br>Bone marrow cells were flushed in FACS buffer (1× PBS containing 0.5% BSA and 2 mM EDTA). Lineage-negative HSPCs were isolated from the tibia and femoral marrow compartments by depletion of lineage-positive cells using a Lineage Cell Depletion Kit (Catalog # 559971 BD Pharmingen) according to the manufacture's protocol.
Instrument	BD LSRFortessa cell analyzer, BD FACSCanto II analyzer, BD LSRFortessa X-20 flow analyzer.
Software	FlowJo
Cell population abundance	The abundances of hematopoietic stem and progenitor cells, the lineage positive cells in bone marrow/spleen/peripheral blood are variable between different experiments, please refer to particular experiments.
Gating strategy	Bone marrow/spleen/peripheral blood lineage positive cell gating:<br>1. Cells were identified by FSC-A/SSC-A gating;<br>2. Singlets were gated by FSC-H/FSC-A;<br>3. Live cells were gated by excluding the PI positive cells;<br>4. Lineage markers: Erythroid cells: TER119+; Granulocytes: Gr1+/Mac1+; Monocytes/Macrophages: Gr1-/Mac1+; B cells: B220+; T cells:CD3e+<br>Lineage negative cells for hematopoietic stem and progenitor cells gating:<br>a. Cells were identified by FSC-A/SSC-A gating;<br>b. Singlets were gated by FSC-H/FSC-A;<br>c. Live cells were gated by excluding the PI positive cells;<br>d. LSK cells were defined as Lin- c-kit+ Sca1+. LK cells are Lin- c-kit+ Sca1-. LS cells are Lin- c-kit- Sca1+.<br>e. LT-HSC: Lin- c-kit+ Sca1+ CD34- CD135-;<br>ST-HSC: Lin- c-kit+ Sca1+ CD34+ CD135-;<br>MPP:Lin- c-kit+ Sca1+ CD34+ CD135+;<br>SLAM-LSK:Lin- c-kit+ Sca1+ CD34- CD150+.

- [x] Tick this box to confirm that a figure exemplifying the gating strategy is provided in the Supplementary Information.
